# In Silico Characterization of the Binding Modes of Surfactants with Bovine Serum Albumin

**DOI:** 10.1038/s41598-019-47135-2

**Published:** 2019-07-23

**Authors:** Osita Sunday Nnyigide, Sun-Gu Lee, Kyu Hyun

**Affiliations:** School of Chemical and Biomolecular Engineering, Pusan National University, Busan, 46241 Korea

**Keywords:** Computational biophysics, Computational models

## Abstract

The binding interactions of the surfactants: anionic sodium dodecyl sulphate (SDS), cationic cetyltrimethylammonium bromide (CTAB), non-ionic octyl glucoside (OG), and zwitterionic 3-[Hexadecyl(dimethyl)ammonio]-1-propanesulfonate (HPS), with bovine serum albumin (BSA) were investigated by computer simulation. The results disclosed that the surfactants bound stably between hydrophobic subdomain IIA and IIIA where tryptophan-213 residue, an important intrinsic fluorophore in BSA is housed. The interactions of the surfactants with the BSA were electrostatic and hydrophobic interactions. The head-groups of SDS, HPS and OG formed hydrogen bonds with the BSA, while that of CTAB was shielded from intermolecular hydrogen-bonding due to intervening methyl groups. Subsequently, molecular dynamics (MD) simulation of the protein-surfactant complexes revealed that hydrogen bonds formed by OG were stronger than those of SDS and HPS. However, the decomposed force-field energies showed that OG had the least interaction energy with the BSA. In addition to MD simulation, it was found by density functional theory (DFT) that the differences in the coulomb interaction energies can be attributed to charge distribution in the surfactants. Overall, free energies calculated by linear interaction energy (LIE) proved that the binding of each surfactant was dominated by differences between van der Waals interactions in bound and free states.

## Introduction

Surfactants are amphiphilic compounds composed of hydrophobic hydrocarbon tails attached to polar hydrophilic heads^1–3^. They are generally grouped into four distinct categories based on the chemical nature of their polar head-groups: cationic (positively charged), anionic (negatively charged), non-ionic (non-charged) and zwitterionic (oppositely charged)^1–11^. When surfactants interact with proteins, their hydrophobic tails bind with the hydrophobic amino acid residues of the proteins while their polar head-groups interact with the proteins’ polar amino acid residues. Previous studies have reported that surfactants interact with proteins specifically and non-specifically depending on the reaction stoichiometry and pH^1,2^.

Bovine Serum Albumin (BSA) is an abundant plasma protein and a universal bio-reagent used in numerous applications, such as enzyme linked immunosorbent assays and immunofluorescence microscopy^2,4,9^. The crystal structure of BSA consists of three homologous domains, each in turn is divided into sub-domain A and B^6^. As a globular protein, BSA has well characterized binding sites and has been found to bind to different types of surfactants, drug molecules and biologically relevant substances^1–6^. When surfactants interact with the BSA protein, they modify the structure of the protein which affects the interactions of the protein with pharmaceutical substances (e.g. drugs)^9^. The encapsulation of these pharmaceutical substances in surfactants or polymer/surfactant mixtures modulates their release. However, the mode of interaction and the binding for BSA-surfactant complex vary from one surfactant type to another^2,4^. The interactions of BSA with surfactants have been studied by various experimental techniques in attempt to gain an in-depth knowledge of the structural modifications occurring at each binding stage^1^. Some of the experimental methods used include microscopy^7^, spectroscopy^2,8,9^ and small angle scattering^1^. Though the binding strengths of various categories of surfactants with BSA have been identified^4,8^, the balance of the driving force is unclear. In other words, whether BSA binding interactions with the surfactants is dominated by hydrogen bonding or other non-covalent interactions, and the contributions of electrostatic and van der Waals forces have not been properly addressed. Adequate atomic/molecular-level interpretation of protein-surfactant interactions is crucial for understanding of a variety of chemical and biochemical phenomena, such as drug delivery and cosmetic preparations^10–12^. On the other hand, obtaining such information from experiments is complex and poses tremendous challenge. Therefore, we seek to use computer simulation to provide atomic-level interpretation of protein-surfactant interactions by decomposing the binding site residues for the protein-surfactant complexes, and the molecular mechanics energies.

In order to predict the ligand binding sites in proteins, the most common approach used is molecular docking^13–20^. Molecular docking often combines a conformational search method and a scoring function to assign the binding affinity for a ligand^21^. The protein is often docked as a rigid molecule and the ligand flexible^21^. Although, in some docking programs a soft scoring function is used to account for the flexibility of the receptor side chains^21^. Owing to the limited capability of the conformational search methods and the scoring functions, the current trend is to carry out MD simulation of a docked protein-ligand complex in order to validate the ligand stability and the molecular interactions^21–25^. MD simulation overcomes the restrictions in movement imposed on the protein in molecular docking, by treating the protein and the ligand as flexible molecules, and thus enabling for an induced fit in the protein-ligand binding^25^. Furthermore, the role of explicit water molecules can be investigated precisely and more accurately^25^. Most of the MD simulation methods combine molecular mechanics energy and solvation models to assess the protein-ligand complex as predicted by docking programs. Subsequently, the interaction energies which are used to quantify the binding strength of the ligand are extracted from the MD trajectories^21–24^. Overall, the use of molecular docking and MD simulations for the efficient screening of a large set of ligands and for the refining of the structures of the final complexes is a rational way to accurately study protein-ligand interactions^25^.

The objective of this study is to investigate the molecular mechanism of protein-surfactant binding interactions, using four major categories of surfactants (i.e. anionic, cationic, non-ionic and zwitterionic surfactants) to provide wide-ranging chemistries and various combination of physical interactions. The binding site residues observed in docking and the molecular mechanics energies (coulomb + van der Waals) calculated from MD simulations were analyzed. The role of water solvent was quantified by binding free energies calculations using linear interaction energy (LIE) approach. To correlate interaction energies with the molecular structure of the surfactants, density functional theory (DFT) calculations of the free and bound surfactant molecules were carried out to estimate the effect of the head-group and tail-group partial charges.

## Simulation Protocol

### Preparation of BSA, SDS, CTAB, OG and HPS structure and co-ordinate files for docking

**Figure 1:**
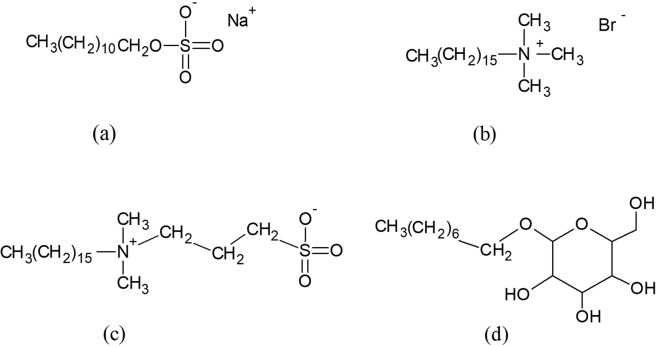
Chemical structure of (**a**) the anionic surfactant, sodium dodecyl sulphate (SDS), (**b**) the cationic surfactant, cetyltrimethylammonium bromide (CTAB), (**c**) the zwitterionic surfactant, 3-[Hexadecyl(dimethyl)ammonio]-1-propanesulfonate (HPS) and (**d**) the non-ionic surfactant, octyl glucoside (OG).

BSA structure file (PDB ID: 4f5s)^26^ was downloaded from protein data bank website (https://www.rcsb.org/) which represents a well-defined high-resolution x-ray structure of the protein^27^. A monomeric *chain B* of the BSA was extracted and used for the entire simulation studies. Water molecules used for crystallization of the molecular structure were removed to leave a clean protein^27^. The surfactants structure files were taken from PubChem website (https://pubchem.ncbi.nlm.nih.gov/). All structures were checked for consistency using PyMOL software. The chemical structures of the surfactants are provided in Fig. 1.

### BSA binding site prediction

**Figure 2:**
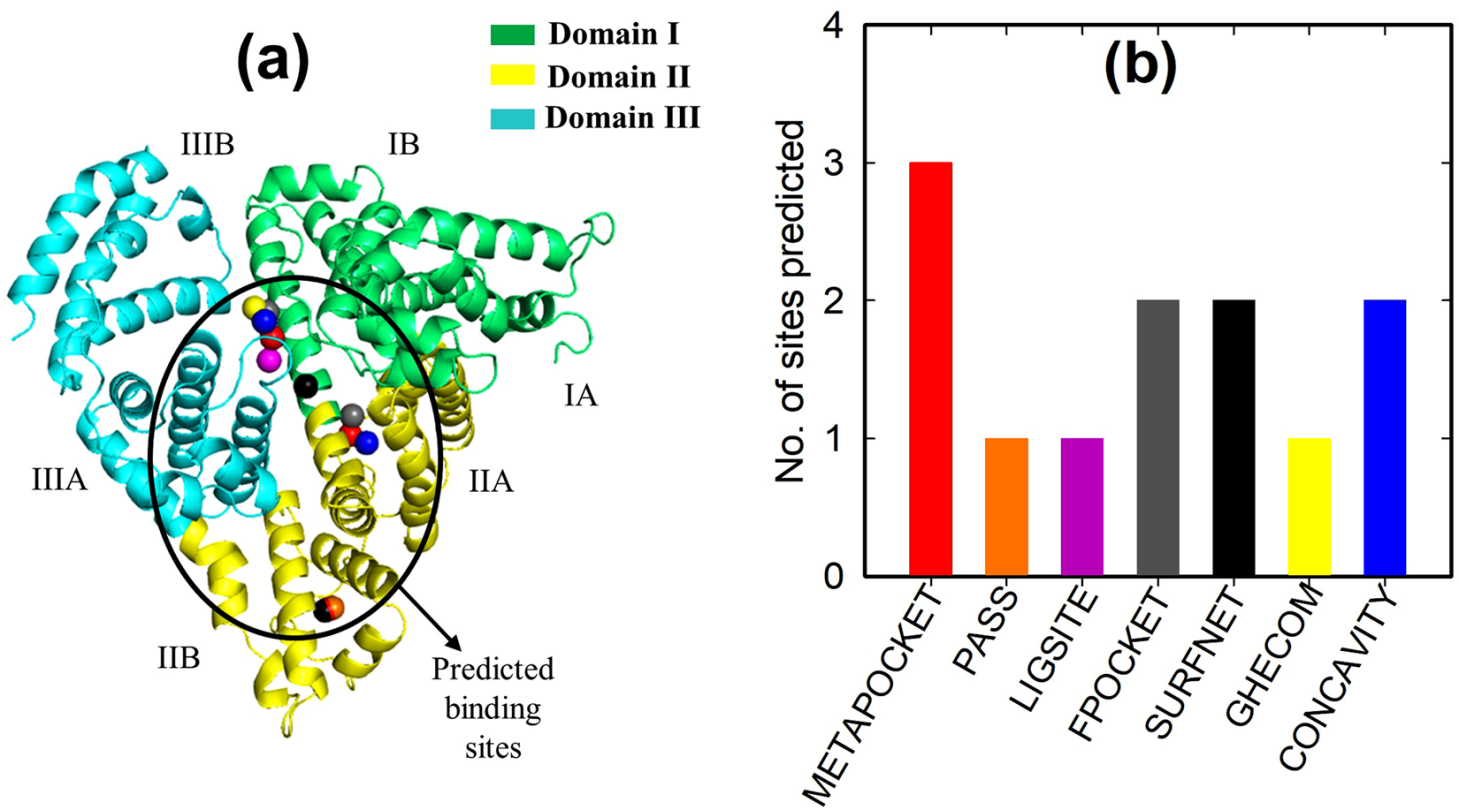
(**a**) Cartoon representation of the BSA monomer structure showing three domains of which each is divided into sub-domain A and B. The binding sites depicted in coloured small spheres were predicted by METAPOCKET^28^, and (**b**) number of predictions versus predictor algorithm.

In order to avoid blind docking and improve docking accuracy, the top three ranked binding sites of the BSA (Fig. 2a) were first determined using METAPOCKET version 2.0^28^. METAPOCKET is a Meta server and a consensus method to identify ligand binding sites on protein surface^28^. METAPOCKET combines the prediction of binding sites from eight methods: LIGSITE, PASS, Q-SITEFINDER, SURFNET, FPOCKET, GHECOM, CONCAVITY and POCASA to improve the overall prediction success rate^28^. Figure 2b shows the number of predictions versus the predictor algorithm. The colors correspond to the predicted binding site in Fig. 2a (i.e. the spheres of various colors). As can be seen, only Q-SITEFINDER and POCASA algorithms failed to identify a binding site within the top three predictions.

### Final molecular docking procedure

The final docking experiments were performed using the docking software AutoDock Vina^19^ with the help of AutoDock Tools (ADT, molecular graphics laboratory). The PDBQT files for the protein and the surfactants were generated using ADT while the docking was done with AutoDock Vina. In generating PDBQT files for the surfactants and the protein, non-polar hydrogens were merged, polar hydrogens were added, and the rotatable bonds were selected for the surfactants. The protein was docked as rigid and the surfactants as flexible. AutoDock Vina generates different ligands conformers using a Lamarkian genetic algorithm (LGA)^19,29^. The GA is implemented with an adaptive local method search^19,29^. The energy-based scoring function includes terms accounting for short range van der Waals and electrostatic interactions, loss of entropy upon ligand binding, hydrogen bonding and solvation^29^. The docking was carried out by setting the grid size to cover the predicted binding sites, using 20 × 28 × 46 Å along x, y, z axes with a grid spacing of 1.0 Å. The grid centre was set at 61.5, 26.4 and 90.1 Å in the x, y and z directions.

### Molecular dynamics (MD) simulation procedure

The top two ranked poses predicted by docking were used for topology generation and subsequent MD simulations. The GROMACS topology files for BSA were generated using GROMACS software but for the surfactants, GlycoBioChem PRODRG server was used to generate the topology files for the GROMOS force field^30,31^. We note that force-field parameters generated by PRODRG server have been used widely without further validation. However, it was shown recently that while the bonded parameters and atom types assigned by PRODRG server are usually correct, the partial charges are largely inaccurate^14,32–35^. In effect, we followed two validation processes to ensure correct partial charges were assigned to the surfactants. First, partial charges on each surfactant was calculated using density functional theory (DFT) at B3LYP level of theory and 6–311 + + G** basis set (discussed in detail later). Second, the calculated partial charges were compared with those given for similar functional groups in GROMACS amino acid residue file since all atoms contained in the surfactants (excluding counter ions) are standard protein atoms.

### Final molecular dynamics production

Molecular dynamics simulations were performed in explicit water solvent. The protein-ligand complexes were constructed following the method previously described^14^. The GROMACS open source software version 5.0.2 and GROMOS96 53a6 force field were used for the entire simulation. A cubic box type was used with periodic boundary conditions applied in all three directions of the box^27^. The distance between the solute and the box was 1 nm^27^. The solution neutrality was maintained with 0.150 M sodium chloride concentration. Energy minimization of the system was performed in two steps, first with steepest descent, followed by conjugate gradient method. Short-range non-bonded interactions were calculated by applying 1 nm cut-off radii, while long-range electrostatic interactions were calculated with Particle-Mesh Ewald (PME) algorithm. The simulations were performed for the systems at 298 K and 1 atm using the NVT and NPT canonical ensembles for equilibration to maintain constant temperature and pressure. The constant temperature was maintained by velocity rescaling temperature coupling thermostat to reproduce a correct kinetic ensemble. The pressure was kept at 1 bar according to the NPT ensemble regime using Parrinello-Rahman barostat^27^. Temperature and pressure couplings were carried out for protein/surfactant and water/ion groups to avoid energy fluctuations that arise from separate coupling of species with few atoms. The LINCS algorithm was applied to constrain all bonds. In both equilibrations steps all heavy atoms were restrained to allow proper distribution of solvent. The duration of pressure equilibration was enough to reach a constant density of approximately 1,000 kgm^−3^ which is the expected density of SPC/E water model and a replica of the experimental value. The final MD production was carried out for 100 ns using leap-frog integrator. A time step of 2 fs was used. The trajectory was stored every 2 ps.

### Binding free energy calculations

The binding free energy for each ligand was calculated using linear interaction energy (LIE) approach^22^. The LIE governing equation for estimation of binding free energy is as follows:

1 $${\rm{\Delta }}{G}_{bind}=\beta \langle {\rm{\Delta }}{U}_{l-s}^{el}\rangle +\,\alpha \langle {\rm{\Delta }}{U}_{l-s}^{vdw}\rangle +\gamma $$

where $\langle {U}_{l-s}^{el}\rangle$ and $\langle {U}_{l-s}^{vdw}\rangle$ are the average ligand-surrounding electrostatic and van der Waals energies collected from two physical states (i.e. the free ligand in water ${\langle {U}_{l-s}^{el}\rangle }_{f}$ and the bound ligand in the solvated protein ${\langle {U}_{l-s}^{el}\rangle }_{b}$). Scaling factors β and α were obtained by fitting experimental and estimated binding free energies for a series of chemically related compounds (Supporting Information Table S1). The parameter γ was set to zero^36,37^.

To use the above LIE equation, we performed MD Simulations of the free ligands in water. Key parameters (Eq. 1) for calculating binding free energy using LIE approach were estimated from MD simulations of free ligands in water. The conditions for MD simulations of free ligands in water are the same as that of the bound ligands to protein (as discussed previously) but without restraint^14^. Since the PME algorithm used for all simulations generates non-decomposable energy, the trajectories obtained from the simulations were re-run with reaction-field-zero for the treatment of long-range electrostatics.

### Partial charge calculations for free and bound ligands

To correlate interaction energies with the electronic structure of the surfactants, we calculated the charge distribution in the free and bound surfactants. For the bound surfactants, calculations were performed for amino acid residues and water molecules at ≤3.5 Å from the surfactants. Configurations were taken from structures that have been energy minimized by MD simulation. The final configurations including hydrogen atoms had 211 atoms (for BSA-SDS), 237 atoms (for BSA-CTAB), 197 atoms (for BSA-OG) and 380 atoms (for BSA-HPS). Calculations were performed on a 16-core desktop server using GAMESS-US software. For all systems, a DFT at B3LYP/6–311 + + G** level of theory was used. The B3LYP uses the generalized gradient approximation (GGA) for the exchange-correlation energy. Each atom was assigned a partial charge by fitting charges to the electrostatic potential (ESP) after obtaining an equilibrium geometry.

## Results

### Validation of the docking/MD simulation protocols

The holy grail of molecular docking is to predict the right pose or conformation of a ligand in a protein’s binding site. This is customarily assessed by superimposing the docked conformation over the experimentally determined crystal structure, and then assessing the root-mean-square deviation (RMSD). However, not all protein-surfactant complexes have been resolved owing to the experimental difficulties, which on the other hand justifies the growing popularity of molecular docking. Fortunately, the crystal structure of the globular protein of the lipocalin family, bovine beta-lactoglobulin complex with SDS has been resolved (LGB, isoform A), which has protein data bank (PDB) ID of 4IB8^10^. First, to calibrate the simulation protocol discussed above, and to investigate the reliability of the docking program, we used the AutoDock Vina software to perform a test docking of SDS binding to LGB, which was followed by 100 ns MD simulation of the highest ranked LGB-SDS complex. Subsequently, the SDS structures from docking and MD simulation were compared with the experimental crystal structure.

**Figure 3:**
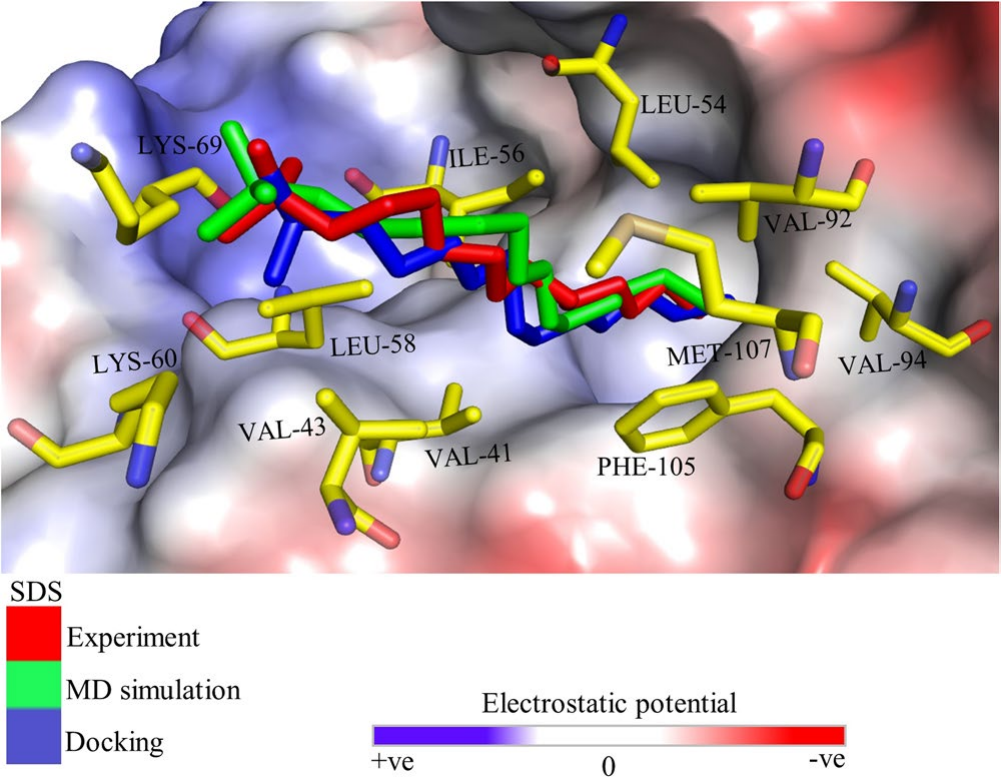
Binding conformation of SDS from molecular docking, MD simulation and experiment. Bovine LGB is shown as EPS surface with residues at ≤3.5 Å of the docked SDS in stick representation.

Figure 3 shows the docked SDS molecule and the one refined by MD simulation, which were superimposed over the experimental structure. Interestingly, we found a good match with the crystal structure judging by the binding pocket prediction. However, the superimposed structure (docking vs experiment) showed high RMSD of 4.0 Å, whereas the subsequent MD simulation of the complex reduced the RMSD to 1.4 Å, which is a reasonable fit. The non-bonded interactions were analyzed by considering LGB amino acid residues at ≤3.5 Å from the docked SDS molecule. The interacting residues at ≤3.5 Å from the docked SDS are similar with those of the experimental structure, and the structure refined by MD simulation. As shown in Fig. 3, no hydrogen bonding interaction was formed between LGB and SDS polar head-group. This agrees with the experimental structure (PDB ID: 4IB8). Also, the MD-simulated structure showed no hydrogen-bonding interaction. However, the SDS polar head-group interacted with LGB polar amino acid residues by electrostatic attraction. The electrostatic potential surface (EPS) revealed that the LGB amino acid residues interacting with the SDS polar head-group are positively charged (i.e. the blue surfaces for LYS-69 and LYS-60). In agreement with previous studies, Loch *et al*.^10^ found that in the structure of LGB isoform complex with SDS and several structures with other ligands, LYS-69 is too far from ligand polar group to contribute to hydrogen bonding. Instead, it formed hydrogen bond with GLU-62 (not shown in Fig. 3) from either the same or neighboring molecule. On the other hand, 9 uncharged hydrophobic amino acid residues interacted with SDS hydrophobic tail, which also agrees well with the docking of SDS in β-barrel binding site of LGB by Bello *et al*.^12^. The authors found that a large number of hydrophobic contacts were made between aliphatic chain of SDS and interior of the binding pocket, as evidenced in the crystal structure (PDB ID: 4IB8)^12^. Therefore, based on the good agreement with experimental studies, the binding of BSA with SDS and other surfactants were investigated by molecular docking and MD simulation, and discussed in the following sections.

### Binding interaction of SDS, CTAB, OG and HPS with BSA

To predict the BSA-surfactants binding interactions and binding site residues, 9 possible docked conformations of the complexes were modeled out of which the top two ranked conformations were initially assessed by MD simulation in order to validate the docking results. The binding conformation of all ligands and the interaction energies of the top two ranked conformations are shown in Supporting Information Fig. S1 and Table S2. It immediately follows from this table that the ligand structures ranked number one had higher interaction energies than the structures ranked number two. In view of this, the structures ranked number one were chosen for further discussion in this study.

**Figure 4:**
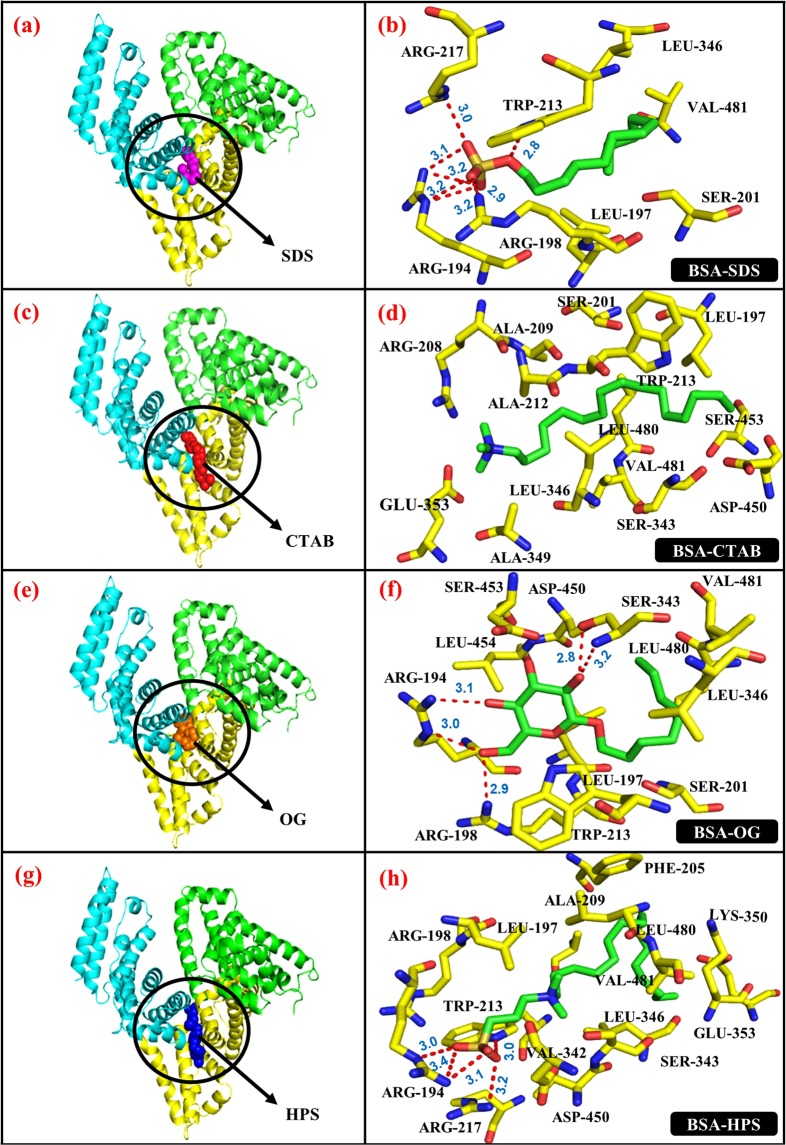
Binding conformation and binding site residues (**a**,**b**) BSA-SDS complex, (**c**,**d**) BSA-CTAB complex, (**e**,**f**) BSA-OG complex and (**g**,**h**) BSA-HPS complex. For ligand binding site location, the BSA is shown as cartoon and the surfactants as spheres and are pointed with arrow. For the binding site residues, the BSA residues and surfactants are in stick representation. The surfactants are shown in conventional CPK colouring. Hydrogen bonds are shown in dotted red lines. The images were created using PyMOL software.

In Fig. 4, the amino acid residues at ≤3.5 Å of the binding pockets for the surfactants are shown. All hydrogen atoms are hidden for clarity. The choice of 3.5 Å cut-off distance is to examine conveniently the non-bonded interactions such as hydrophobic and electrostatic interactions between the surfactants and the protein. As can be seen, the polar head-groups of the surfactants interacted with various BSA polar amino acid residues, while their hydrophobic tails bound closely to non-polar BSA amino acid residues.

In the case of SDS (Fig. 4a,b), the binding occurred between hydrophobic subdomain IIA and IIIA where TRP-213 residue is housed, which is a major binding site of the BSA (Fig. 2a). The SDS head-group was attracted to positively charged arginine residue side chains. Some of the BSA residues and SDS head-group oxygen atoms interacted by regular and bifurcated hydrogen bonding as summarized in Table 1. The uncharged hydrophobic tail of SDS interacted with the BSA hydrophobic residues namely: LEU-197, LEU-346, and VAL-481. The presence of a polar amino acid residue (e.g. SER-201) at the SDS tail may suggest intramolecular interaction with neighboring polar residues, which is notably the case with other surfactants.

**Table 1:** Number of hydrogen bonds (HBs) formed by the surfactants’ polar head-groups with BSA amino acid residues and the average HB distance.

BSA-surfactant complex	Interacting BSA residues	No. of bifurcated HBs	No. of regular HBs	Total no. of HBs	Avg. HB dist. (Å)
BSA-SDS	Arg 194, Arg 198, Arg 217, Trp 213	3	1	7	3.06
BSA-CTAB	Glu 353, Arg 208	—	—	—	—
BSA-OG	Arg 194, Arg 198, Ser 343	1	3	5	3.00
BSA-HPS	Arg 194, Arg 217, Trp 213	2	1	5	3.14

The binding conformation and binding site residues for CTAB are somewhat like SDS, although it extended to domain IIB (Fig. 4c,d). CTAB head is bound between oppositely charged ARG-208 and GLU-353. It can be seen that the CTAB nitrogen atom is shielded from intermolecular hydrogen bonding with the BSA, presumably due to the intervening methyl groups (Fig. 4d). As reported by Loch *et al*.^10^, a major role of hydrophobic interactions in ligand binding appeared to prevail even with the repulsion between positively charged CTAB and ARG-208 residue located at the binding site entrance. Unlike SDS, TRP-213, an important intrinsic fluorophore in the BSA is closer to the CTAB tail than the head. Various hydrophobic amino acid residues (ALA-209, ALA-212, ALA-349, LEU-197, LEU-346, LEU-480 and VAL-481) interacted strongly with CTAB hydrophobic tail.

The binding conformation and binding site residues for OG are shown in Fig. 4e,f. OG formed 5 hydrogen bonds with arginine and serine residues (Table 1). TRP-213 appeared to be close to the glucoside carbon ring, whereas other hydrophobic residues of the BSA are distributed between OG tail (LEU-346, LEU-480 and VAL-481) and the carbon ring (LEU-197 and LEU-454).

For zwitterionic HPS (Fig. 4g,h), the binding also occurred in the same binding site as other surfactants but extended to domain IIB as CTAB. HPS has oppositely charged head-group (sulfonate anion and quaternary ammonium cation), and formed 5 hydrogen bonds with various arginine residues, and with TRP-213 (Table 1). In addition, the benzene ring of TRP-213 is close to positively charged HPS nitrogen and may interact by cation-pi. However, as observed with CTAB, the polar head-group atoms in the ammonium cation were shielded from intermolecular hydrogen bonding with the BSA, while the tail-group of the surfactant bound closely to various hydrophobic residues (LEU-480, LEU-346, LEU-197, ALA-209, VAL-481, VAL-342 and PHE-205).

### Stability of the docked complexes

**Figure 5:**
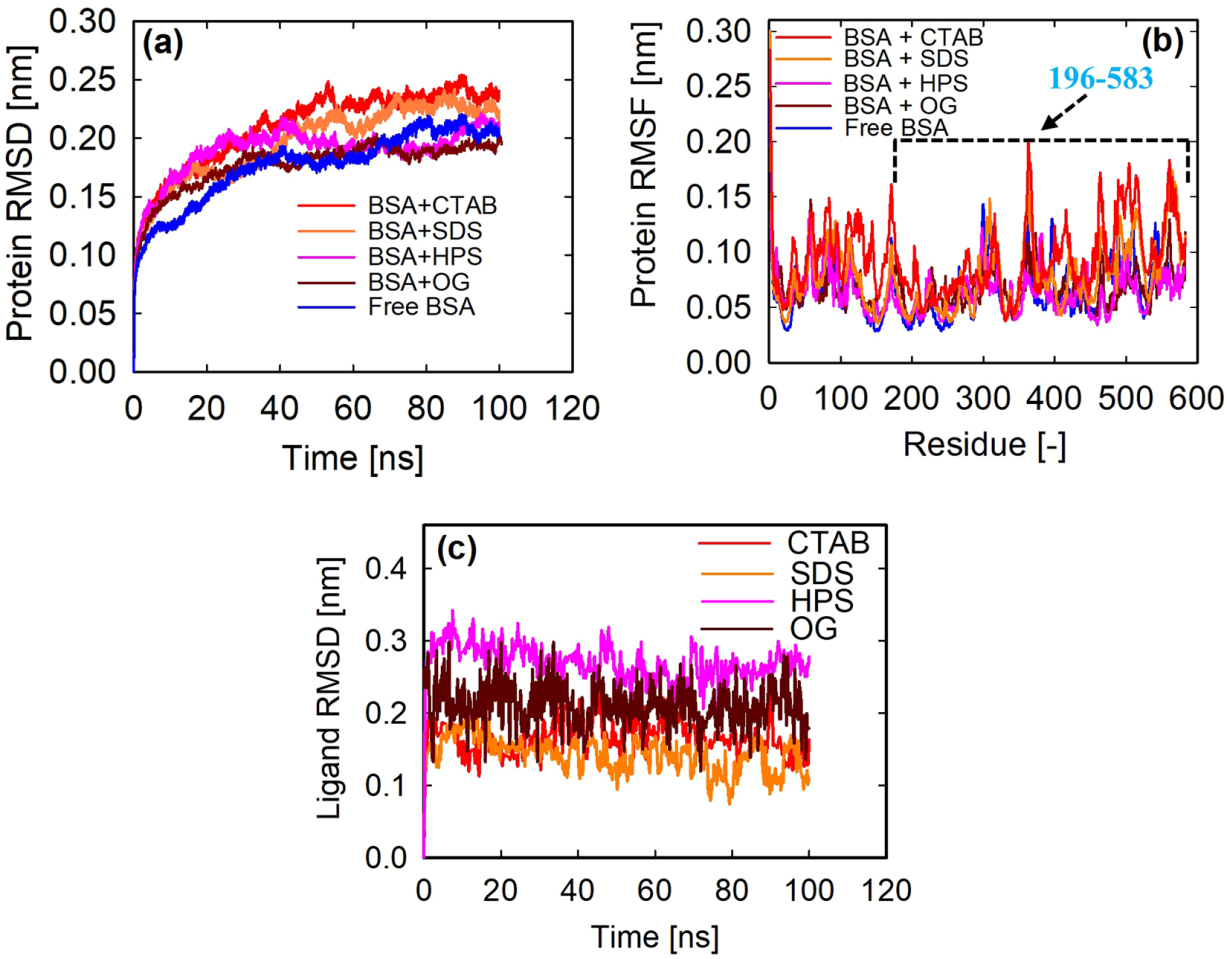
(**a**) Root-mean-square deviation (RMSD) of the protein backbone atoms as a function of simulation time, (**b**) root-mean-square-fluctuation (RMSF) per residue over the 583 amino acid residues, the arrow points to approximately domains II and III comprised of 196–583 residues and (**c**) Ligand RMSD as a function of simulation time.

Each of the BSA-surfactant complex discussed above was subjected to 100 ns MD simulation. The structural fluctuation or stability of each complex was measured by the root-mean-square-deviation (RMSD) and the root-mean-square-fluctuation (RMSF). The RMSD indicates the average displacement of the atoms at an instant of the simulation relative to the crystal structure^38^. The crystal (or reference) structure was taken as the first frame of the simulation. The RMSDs of the protein backbone atoms were analyzed to investigate if the system reached equilibrium. It can be seen in Fig. 5a that all systems reached equilibrium at 40 ns. That is, their RMSDs increased rapidly from 0 to 20 ns, after which it increased smoothly to 40 ns and then remained steady. Subsequent analysis of interaction energies was carried out using trajectories from 40 to 100 ns of the MD production phase. The free BSA which is not required for the protein-surfactant interaction energy calculation is shown for comparison with the surfactant-bound structures. Subtle differences in RMSDs after reaching equilibrium may suggest that the stability of the BSA was altered slightly upon surfactants binding, with the ionic surfactants (i.e. SDS, HPS and CTAB) showing higher average RMSD values than the non-ionic OG.

The RMSF per residue calculated over the 583 amino acid residues is shown in Fig. 5b. RMSF is a measure of the displacement of a particular atom, or group of atoms, relative to the crystal structure, averaged over the number of atoms^38^. It can be seen from this figure that the domain 1 of the BSA (i.e. residues 1–195) was quite stable while most of the fluctuations occurred between domains II and III (i.e. residues 196–583) which contained the major binding site residues for the surfactants.

### Ligand stability and hydrogen bond kinetics

**Figure 6:**
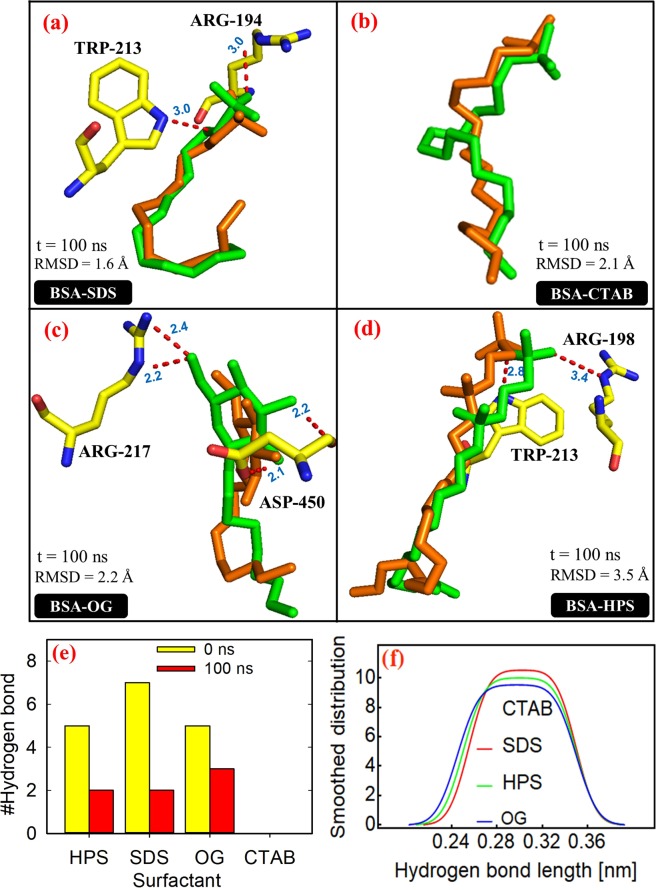
Superimposed structures of the surfactants showing hydrogen bonds (dotted red lines) from a single MD snapshot at 100 ns (**a**) BSA-SDS (**b**) BSA-CTAB (**c**) BSA-OG and (**d**) BSA-HPS. The BSA amino acid residues and surfactants are shown in stick representations with the docked surfactant in orange and MD-simulated surfactant in green. All hydrogen atoms are hidden for clarity; (**e**) average number of hydrogen bonds and (**f**) hydrogen bond distribution as a function of bond length.

We investigated the persistence of the ligands in the binding site and their hydrogen bond networks with the protein over the 100 ns MD production phase. First, the RMSDs of the ligands were calculated by aligning the MD-simulated structures to the initial structures, and second, the stability of their hydrogen bonding interactions was analyzed. Figure 5c shows that the RMSDs of the ligands from their reference structures are minimal and are in the same range as that of the protein. This observation is well supported by the superimposed structures (docking vs simulation) shown in Fig. 6(a–d). However, the number of hydrogen bonds at 100 ns decreased when compared with the values predicted in docking, which may be due primarily to the presence of some stable water molecules in the binding site (discussed later), and also the observed RMSDs of the ligands from their initial structures. The number of hydrogen bonds formed by OG at 100 ns is higher than those of SDS and HPS (Fig. 6(a–d)), which agrees with the average number calculated over the entire trajectory (Fig. 6e). Figure 6(a–e) indicates that no hydrogen bonding interaction exists between CTAB and the BSA which is consistent with the docking studies. Interestingly, the average OG hydrogen bond length at 100 ns appeared significantly shorter (or stronger) than that observed in docking. That is, on the average, the bond length for OG is 2.23 Å when compared with SDS (3.0 Å) and HPS (3.1 Å), which has direct implication on the interaction energies. This finding is further confirmed by the hydrogen bond distribution calculated over the entire trajectory which is shown in Fig. 6f. The distribution is shifted towards smaller hydrogen bond length for OG, and the mean of the distribution is 2.8 Å for OG, 3.06 Å for SDS and 3.02 Å for HPS. The discrepancy in the bond length between the distribution mean and that calculated at 100 ns indicates that a single snapshot or the total number of bonds is insufficient to characterize the energetics of the protein-ligand hydrogen bonds. In view of this, we evaluated the hydrogen bond lifetimes and relaxation times using the Luzar and Chandler theory of hydrogen bond kinetic^39^, and the thermodynamics as described by van der Spoel *et al*.^40^.

According to Luzar and Chandler theory^39^, the formation of a hydrogen bond is considered to be an equilibrium process, and the free energy of hydrogen bond formation is calculated from the corresponding equilibrium constant^40^. First, the lifetime of a hydrogen bond is calculated from the rate constants of hydrogen bond breaking and re-forming, and its distribution is converted into an autocorrelation function. Second, the autocorrelation function is fitted exponentially and then integrated to obtain the hydrogen bond relaxation time^41^. The calculated kinetic and thermodynamic parameters are shown in Table 2. As can be seen from this table, an average BSA-OG hydrogen bond has longer lifetime and slower switching dynamics (or longer relaxation time) in comparison with those of BSA-SDS and BSA-HPS. Also, the free energy of breaking an average BSA-OG hydrogen bond is higher than those of BSA-SDS and BSA-HPS. Based on Table 2, the strength of the ligand hydrogen bonds increased in the order: OG > HPS > SDS.

**Table 2:** Kinetics and thermodynamics of protein-ligand hydrogen bonds at 298 K.

Surfactant molecule	Lifetime (ps)	Relaxation time (ps)	Free energy (kJ/mol)
BSA-SDS	17.40	25.08	12.52
BSA-OG	41.48	328.30	18.89
BSA-HPS	26.61	178.07	17.37
BSA-CTAB	—	—	—

The importance of the kinetics and thermodynamics of the protein-ligand hydrogen bonding interactions is that it helps us rationalize the contribution of hydrogen bonding to the protein-ligand potential energies (coulomb and van der Waals), and also the binding free energies which are discussed in the following sections.

### Energy decomposition for the ligands binding with the BSA

**Figure 7:**
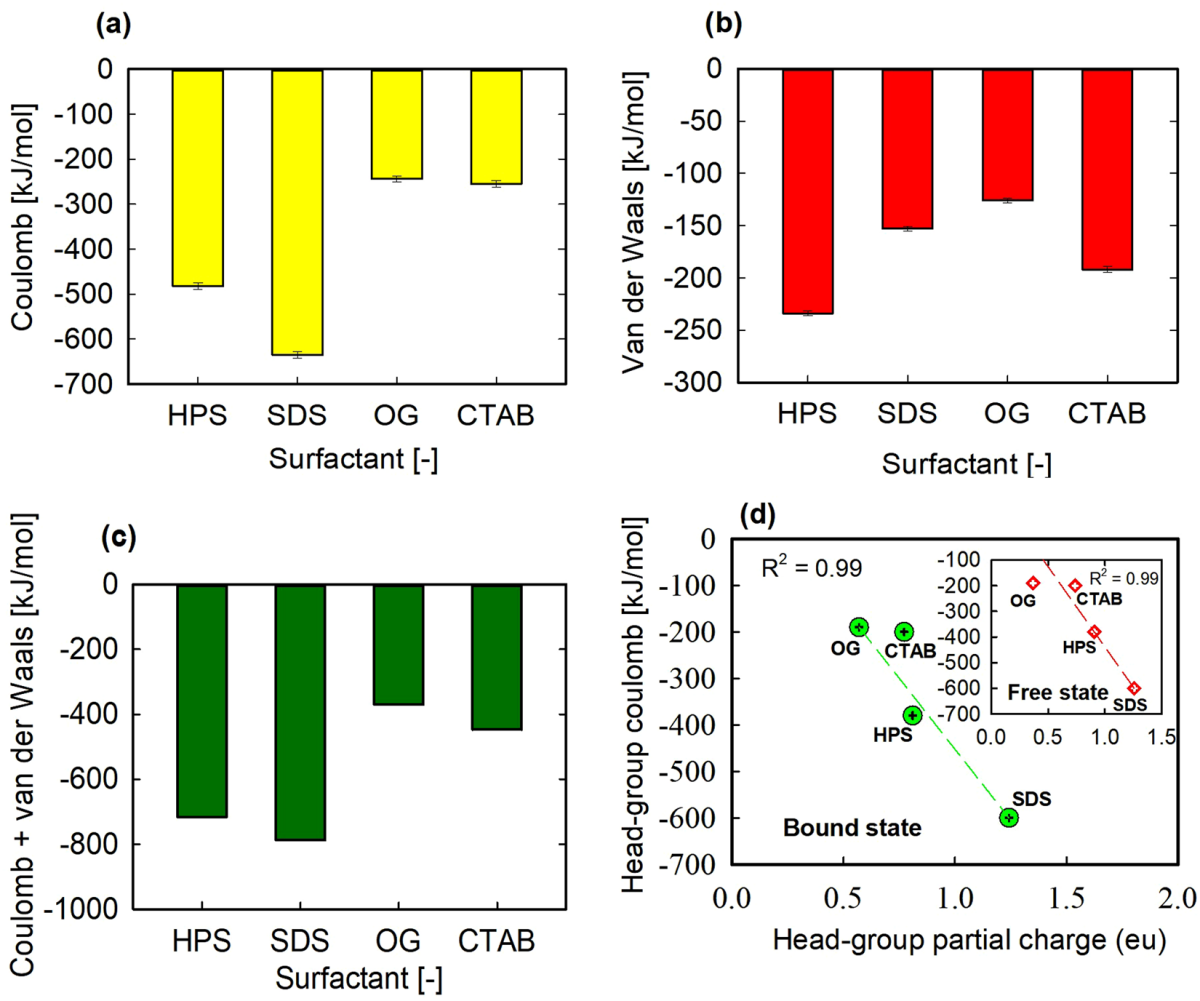
(**a**) Coulomb interaction energy as a function of surfactants, (**b**) van der Waals energy as a function of surfactants, (**c**) total energy (coulomb + van der Waals) as a function of surfactants and (**d**) head-group coulomb interaction energy as a function of the absolute value of the bound surfactants head-group partial charges in electron unit (eu). The inset plot is for the free state of the surfactants. For HPS the average of the oppositely charged head-group partial charges was taken.

Energy decomposition analysis was carried out using energy files from MD simulation trajectories. Excluding the entropic terms and the solvation contributions, the non-bonded interaction energies (electrostatic and van der Waals energies) which are modelled using coulomb and Lennard Jones terms were calculated. The bonded interaction energy between the surfactants and the BSA is zero. The hydrogen bond energy is distributed largely in the coulomb and slightly in the van der Waals energy^32^. As shown in Fig. 7, all energy terms are negative, which is an indication of a net attractive interaction. However, the coulomb energy term increased in the order: SDS > HPS ≫ CTAB ≥ OG (Fig. 7a). The lower coulomb energy for CTAB in comparison with the other ionic surfactants is reasonable since CTAB head-group atoms were shielded from intermolecular hydrogen bonding with the BSA due to intervening methyl head-groups (Fig. 4d), while SDS and HPS formed hydrogen bonds with the BSA. OG had the lowest coulomb interaction energy, though it formed the strongest hydrogen bond with the BSA (Fig. 6 and Table 2). This important finding was explained by DFT calculation (discussed later).

Meanwhile, the van der Waals energy increased in the order: HPS > CTAB > SDS > OG (Fig. 7b). Apparently, the increasing order of the van der Waals energy is in the order of increasing hydrophobic chain length of the surfactants. HPS and CTAB have 16 carbon each in their alkyl tails, but HPS contains several bridging methylene groups sandwiched between the oppositely charged head-group atoms. Although OG has higher number of carbon atoms than SDS, its uncharged alkyl tail is shortened by the presence of a carbon ring (Fig. 1d). Overall, the sum of the interaction energies (coulomb + van der Waals) increased in the following order: SDS > HPS > CTAB > OG (Fig. 7c), which interestingly coincides with the increasing order of their experimentally determined binding free energies with the BSA^4,42–45^.

### Relationship between partial charge distribution in the ligands and their interaction energies

To understand why the ionic surfactants have higher interaction energies with the BSA than the non-ionic OG (despite their lower hydrogen bond strength or the absence of hydrogen bond), we decomposed the coulomb and van der Waals energies into contributions from head-group and tail-group atoms, and then showed how they are related to the partial charges on these group of atoms in their free and bound states. The calculated values of the partial charges are shown in Table 3. For the bound surfactants, calculations of partial charges were performed for amino acid residues and water molecules at ≤ 3.5 Å from the surfactants. It is interesting to note from Table 3 that the atomic partial charges in the free surfactants are distributed largely on the head-group atoms and slightly on the tail, and that the distribution of these charges changed upon binding with the protein owing to various loses and gains in charge density from the solution^46^. The amount of electrons gained increased in the order: OG > CTAB > HPS > SDS (i.e. SDS lost 0.157 electron unit while HPS lost 0.078 electron unit).

**Table 3:** Grouped atomic partial charges for free and bound surfactants calculated by DFT at B3LYP level of theory and 6–311 + + G** basis set^a^.

Surfactant molecule	Head-group	α-group	α-group + alkyl tail	Net charge
**Free surfactants (no solvation effect)**				
SDS	−1.258	+0.300	+0.258	−1
OG	−0.368	+0.198	+0.368	0
HPS	−1.000/+0.812	+0.063	+0.188	0
CTAB	+0.738	+0.044	+0.262	+1
**Surfactants bound to solvated BSA protein**				
SDS	−1.242	+0.418	+0.399	−0.843
OG	−0.570	−0.504	+0.404	−0.166
HPS	−0.971/+0.649	+0.260	+0.400	+0.078
CTAB	+0.772	−0.021	+0.063	+0.835

^a^Huibers^47^ included α group as part of the head-group whereas others^48–50^ included α group as part of the tail-group.

In relation to the potential energies (coulomb and van der Waals), the results plotted in Fig. 7d indicates that the head-group coulomb interaction energy scales linearly with the absolute value of the head-group partial charge in the free and bound states of the surfactants. In the case of the tail-groups, no scaling relationship was found between their partial-charges and coulomb energies, which may be due primarily to the small differences in these charges for the ionic surfactants. Moreover, these charges are all positive which may introduce some repulsive forces considering the binding site dominated by positively charged arginine residues. The van der Waals energy on the other hand scales with the size (not the partial charge) in the tail- or head-group of the surfactants (data not shown). Overall, our finding suggests that the predominant contribution to the coulomb interaction energy of the surfactants is the amount of partial charge that is on their head-group atoms and not the strength of hydrogen-bonding interaction which followed a different trend (i.e. OG > HPS > SDS (Table 2)).

It is important to mention that the systematic means of organization applied to determine which atom belongs to the head- or tail-groups impacts on the calculated values of the head-groups coulomb energies and partial charges (Fig. 7d). The existence of significant amount of partial charge on the α-methylene group atoms preceding the head-groups of the ionic surfactants (e.g. CH_2_ group nearest to O atom in SDS) makes a case for their inclusion as part of the head-groups atoms^47^. Such inclusion reported by Huibers^47^ would alter the head-groups coulomb energies and the increasing order of the calculated head-groups partial charges for the bound surfactants (Table 3). However, several studies favored the inclusion of the α-methylene group as part of the hydrophobic tail-group^48–50^, which is consistent with this study.

### The role of water solvent

**Figure 8:**
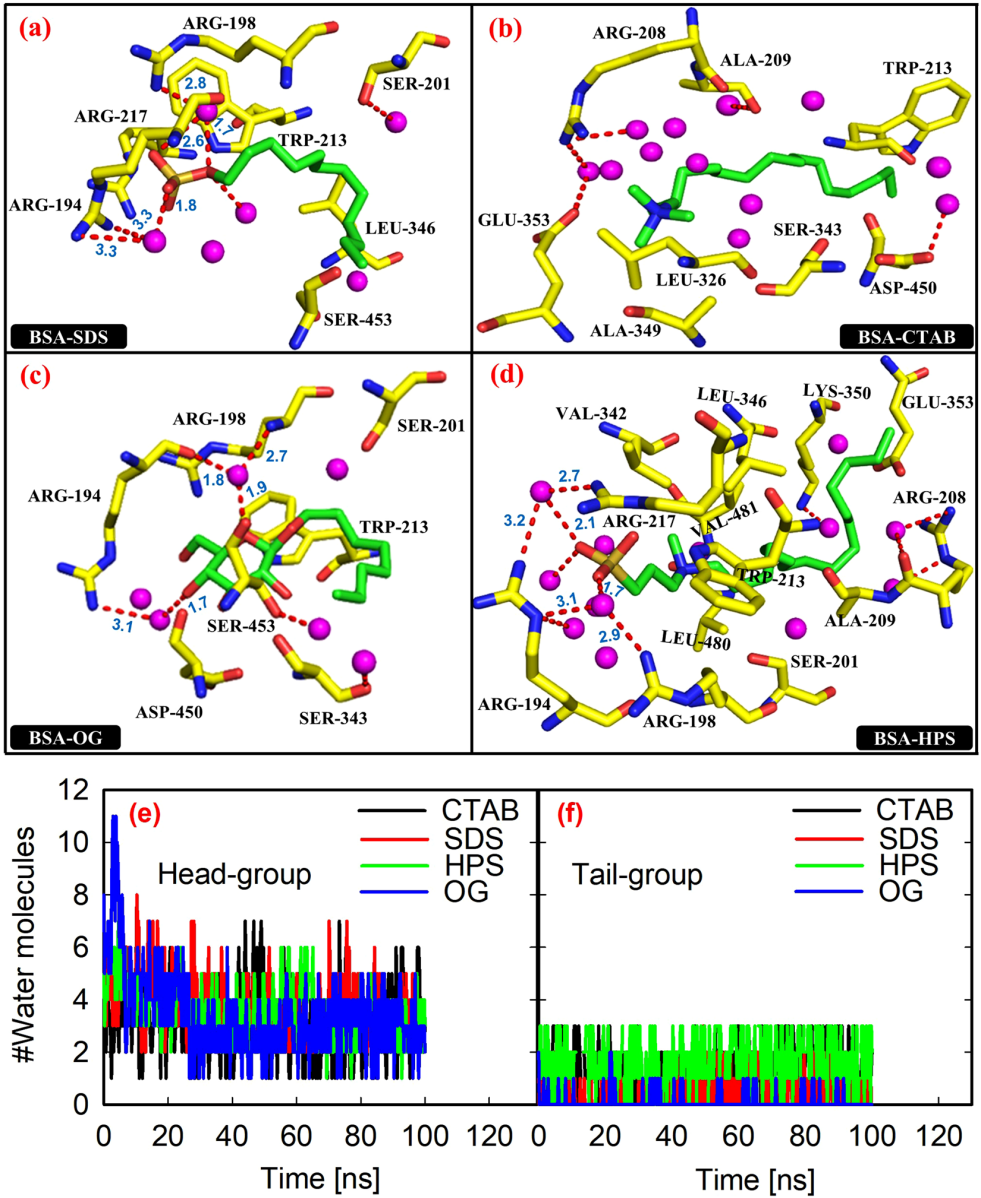
Water-mediated interactions in the model systems, BSA and surfactants are shown in stick representations with the surfactants in CPK colours, and water molecules shown as spheres are coloured in magenta. Water-mediated hydrogen bonds (red dotted lines) are labelled (**a**) BDS-SDS, (**b**) BSA-CTAB, (**c**) BSA-OG, (**d**) BSA-HPS; number of water molecules within 0.35 nm of the surfactants (**e**) head-group and (**f**) tail-group.

Interactions made by water molecules in the systems were analyzed by considering water molecules that are within 0.35 nm of the surfactants (Fig. 8(a–d)). For clarity, all direct hydrogen bonds between the BSA and the surfactants in are hidden. It can be seen from this figure that there are more water molecules close to each surfactant head-group than the tail-group. Except for CTAB, some of the water molecules close to the surfactants head-groups mediate their hydrogen bonding interaction with the BSA by acting as a bridge, owing to their dual ability to act either as donor or acceptor.

Generally, only unstable water molecules are displaced from the binding site into the bulk phase, and to better understand the desolvation process, we calculated the number of water molecules that are within 0.35 nm of the surfactants over the entire simulation window. Calculated values which are shown in Fig. 8(e,f) revealed that some of the water molecules close to the surfactants head-groups were displaced, leaving approximately four stable water molecules on the average. Meanwhile, only an average of one water molecule was close to the tail-groups of the surfactants. The expulsion of water molecules from the hydrophobic pockets of the binding site into the bulk solution provides favourable entropic contribution to the binding free energy, while the water-mediated interactions contribute to the binding enthalpy.

For comparison with the direct protein-ligand hydrogen bonds discussed previously, we calculated the lifetimes, relaxation times and free energies of breaking the water-mediated hydrogen bonds. Calculated values shown in Table 4 indicate that an average BSA-OG water-mediated hydrogen bond is thermodynamically more stable than those of BSA-SDS and BSA-HPS. Also, an average BSA-OG water-mediated hydrogen bond has longer lifetime and slower switching dynamics (or longer relaxation time) than those of BSA-SDS and BSA-HPS. However, for all the surfactants, these bonds are much weaker and less stable than the direct hydrogen bonds.

**Table 4:** Kinetics and thermodynamics of water-mediated hydrogen bonds at 298 K^a^.

Surfactant molecule	Lifetime (ps)	Relaxation time (ps)	Free energy (kJ/mol)
BSA-W-SDS	12.08	12.87	10.86
BSA-W-OG	21.27	61.25	14.73
BSA-W-HPS	14.16	26.93	12.69
BSA-W-CTAB	—	—	—

^a^W indicates water molecule.

### Binding free energy calculation

**Figure 9:**
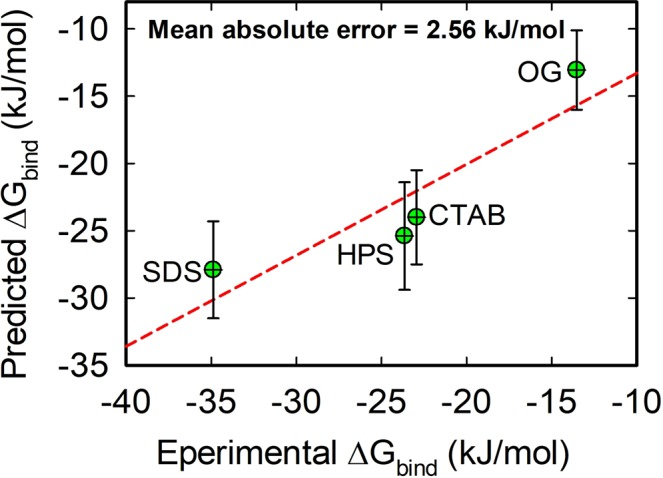
Predicted vs experimental binding free energies using LIE approach.

Ultimately, the determination of the binding free energy is what is required in order to assess the role of water molecules in ligand binding. Thus, using linear interaction energy (LIE) approach the binding free energies of the surfactants were calculated. Based on the MD simulation of the free ligands in water, the electrostatic and van der Waals energies (${\langle {U}_{l-s}^{el}\rangle }_{f}$ and ${\langle {U}_{l-s}^{vdw}\rangle }_{f})$ of the free ligands (Eq. 1) were estimated (see Supporting Information Table S3), while ${\langle {U}_{l-s}^{el}\rangle }_{b}$ and ${\langle {U}_{l-s}^{vdw}\rangle }_{b}$ are plotted in Fig. 7a,b. The scaling factors β, α and γ were assigned values of 0.16, 0.5 and 0 using various chemically related ligands as a training set (Supporting Information Table S1). The scaling factors (β, α and γ) are well known to be system dependent^14,36,37^. Chen *et al*.^37^ assigned γ = 0 for absolute binding free energy calculation while Abdel-Hamid and McCluskey^14^ assigned 3.2 to γ in order to produce a reasonable value for the estimated binding free energy. Calculated values of ${\rm{\Delta }}{G}_{bind}$ were compared with experimentally determined values at neutral pH and 298 K. In the case of SDS and HPS, their binding free energies were calculated from experimentally determined dissociation constants (inverse of association constant), *K*_*D*_, reported by Gelamo and Tabak^44^ using the following equation:

2 $${\rm{\Delta }}{G}_{expt}=RT\,ln{k}_{D}$$

where *R* is gas constant. It is important to mention that experimental binding free energies of the surfactants vary slightly across authors^42–45^, but trends are the same (i.e. SDS > HPS > CTAB > OG). Thus, we averaged ${\rm{\Delta }}{G}_{expt}$ values reported at low surfactant concentration (typically 1–10 times the protein concentration), where surfactants binding to BSA is specific^45^. Figure 9 shows a good agreement between predicted and experimental binding free energies. The values are shown in Table 5 for better comparison. The mean absolute error in the calculated binding free energy for all ligands is 2.56 kJ/mol. It should be noted that errors are not unique to LIE method used in this study but may affect any computational method to calculating a binding free energy where experimental structures for all protein-ligand complexes are not available to be used as the starting structure for MD simulation. The calculated binding free energies were dominated by differences between the van der Waals energies in the bound and free states of the ligands. That is, for all the surfactants, their van der Waals interactions were more favourable in the bound state than they were in the free state, while the electrostatic (coulomb) interactions were less favourable in the bound state.

**Table 5:** Comparison of free energies derived from LIE and experiment at 298 K and neutral pH^a^.

Complex	${\rm{\Delta }}{{\boldsymbol{G}}}_{{\boldsymbol{bind}},\exp }$ [kJ/mol]	${\rm{\Delta }}{{\boldsymbol{G}}}_{{\boldsymbol{bind}},{\boldsymbol{predicted}}}$ [kJ/mol]
BSA-CTAB	−22.94^43,44^	−24.00 ± 3.50
BSA-SDS	−34.89^42,44,45^	−27.90 ± 3.60
BSA-HPS	−23.65^44^	−25.39 ± 4.00
BSA-OG	−13.54 ± 0.44^4^	−13.10 ± 3.10

^a^Calculated value for SDS using association constant reported by Gelamo and Tabak^44^ is 29.36 kJ/mol and values reported by De *et al*.^42^ and Nielson *et al*.^45^ are 32.40 kJ/mol and 42.9 kJ/mol, giving average of 34.89 kJ/mol over all authors.

## Discussion

Proteins interact differently with surfactants at monomeric, bulk and micellar concentrations, and may have different surface activity depending on the protein/surfactant stoichiometry and pH^1,51^. Given the difficulties and limitations in concentration regimes of most experimental techniques, the use of molecular docking and molecular dynamics simulations to investigate protein-surfactant interactions at the atomic/molecular-level have seen a significant increase. Recently Hu *et al*.^13^ investigated the binding of sodium dodecyl sulphate (SDS) to beta-lactoglobulin (LGB) using molecular docking and hydrogen/deuterium exchange mass spectrometry. They proposed two ligand binding sites, first in the β-barrel (internal binding site (Fig. 3)) and the second one on the protein surface in the region where vitamin D3 was previously localized as shown by Yang *et al*.^52^. When compared with our findings for the docking of LGB-SDS, some similarities and differences can be pointed out regarding the SDS binding in the β-barrel site of LGB. For instance, they observed that SDS binding to LGB was driven by electrostatic interaction between the surfactant polar head-group and positively charged lysine residue side chains, whereas the tail-group interacted with LGB hydrophobic amino acid residues. This agrees with the current study. On the other hand, they observed hydrogen-bonding interaction between SDS and LYS-69 in the internal binding site of LGB, and with LYS-138 and LYS-141 residues in the external binding site. Apparently, our result differed in this regard as we observed no hydrogen-bonding interaction which is consistent with the LGB-SDS crystal structure (PDB ID: 4IB8) published recently by Loch *et al*.^10^. Moreover, the authors^10^ showed that in structures of both LGB isoform complexes with SDS and several structures with other ligands, LYS-69 is too far from ligand polar group to contribute to hydrogen bonding. In addition, the absence of hydrogen-bonding interaction in the docked LGB-SDS agrees with the structure of LGB–SDS complex published by Gutierrez-Magdaleno *et al*.^53^.

The binding interaction of various surfactant categories with BSA have also been investigated by several authors using combined molecular docking and experimental techniques. Dasmandal *et al*.^9^ used molecular docking and spectroscopy experiments and found that the binding of anionic amino acid surfactant (AAS) to BSA occurred between hydrophobic subdomain IIA and IIIA where tryptophan-213 residue is located. They observed multiple hydrogen bonding and hydrophobic interactions between the AAS ligand and the BSA which agrees with the current study. Srivastava and Alam^54^ observed in molecular docking that SDS bound to BSA at both domain IIA and IB binding sites, but with greater number of interactions or affinity at hydrophobic subdomain IIA. In addition, several other studies using molecular docking and experiments have identified the hydrophobic subdomain IIA and IIIA as the preferred and the most stable binding sites for various surfactant types and pyrrolidinium based ionic liquids^55–58^. However, the driving force of the binding of various surfactant categories to BSA and the contributions of coulomb and van der Waals interactions have not been adequately addressed, which is the major contribution of the current study.

Therefore, using energy decomposition, the potential energies of interactions in the bound and free states of the surfactants were calculated. In the case of the bound state of the surfactants, we found that coulomb energy dominated the ionic surfactants interactions with the BSA when compared with the non-ionic OG^59^. On the other hand, the van der Waals energy scaled directly with the size of the hydrophobic tail of the surfactants. Evidently, the difference between the coulomb interaction energies of SDS or HPS and OG appeared significantly higher than the difference in their van der Waals energies. The coulomb interaction energy of CTAB with the BSA was only marginally higher than that of OG, presumably because of the smaller difference in their head-groups partial charges in comparison with SDS or HPS, and the absence of hydrogen-bonding interaction. Moreover, CTAB bound between oppositely charged ARG-208 and GLU-353 and would experience some unfavourable electrostatic repulsion from ARG-208.

We have decomposed the coulomb and van der Waals energies into contributions from head-group and tail-group atoms in order to relate them to the partial charge distribution in the surfactants. The results revealed that the head-group coulomb interaction energies scaled linearly with the atomic partial charges on the surfactants polar head-groups which were calculated using DFT. We note, however, that this scaling relationship might be affected by other factors, such as hydrogen bonding interactions and the nature of the binding site residues. In the case of the tail-groups, no scaling relationship was found between their partial-charges and coulomb energies, since the charges in the tail-groups are all positive which potentially introduced some repulsive forces considering the binding site dominated by positively charged arginine residues. Van der Waals energy on the other hand scaled with the size of the tail- or head-groups of the surfactants. Therefore, the greater concentration of atomic partial charge on the ionic surfactants head-groups in comparison with the non-ionic surfactant contributed predominantly to their higher coulomb interaction energies. On the other hand, the relatively strong hydrogen bonds formed by OG with the BSA appeared to contribute significantly to its coulomb interaction energy.

In the case of the free surfactants in water, the van der Waals interactions were more favourable in the bound state than they were in the free state, while the electrostatic (coulomb) interactions were less favourable in the bound state (see Supporting Information Table S3). Using LIE approach, the solvation effect was quantified by calculating the surfactants binding free energies. The calculated binding free energies showed good agreement with experiments. The increasing order of the surfactants binding free energies is the same as the increasing order of the sum of their total potential energies (coulomb + van der Waals) in the bound state. We note that the LIE equation is missing explicit terms for the treatment of entropy. LIE includes conformational entropy implicitly through the linear response expressions which relate potential energy differences to free energy differences^36^. Also, studies have shown that a good correlation exists between hydrophobic effects and van der Waals interactions^22^. Thus, it would appear that entropic contributions and hydrophobic effects are confined in the empirically optimized value of the van der Waals scaling parameter (α)^22,36^. The rationale here is not that hydrophobic effects now reside in the van der Waals energy term, but rather based on the observations that the solvation free energies for typical non-polar compounds scale linearly with solute size measures and that the average van der Waals interaction energies scale approximately linearly with solute size both in polar and non-polar solvents^22,36^. Hence, it is possible to adapt van der Waals interactions to provide a convenient measure of hydrophobic interactions. Therefore, the larger value of α (0.5) compared to β (0.16) indicates a dominance of the van der Waals forces, hydrophobic effects and entropy for the surfactants binding to the BSA. This finding agrees with previous studies^10,60^. Loch *et al*.^10^ showed recently that the specific binding of 12-carbon surfactants by bovine β-lactoglobulin was driven largely by entropy, with small enthalpy contribution. Further, Reynolds and co-workers^60^ reported that the largest contribution to the specific binding of octyl and dodecyl sulfates to the sites of highest affinity of native bovine serum albumin was primarily hydrophobic rather than coulombic interaction.

## Conclusion

The binding of four major categories of surfactants to BSA has been studied by molecular docking and molecular dynamics simulation. The surfactants were found to bind between hydrophobic subdomain IIA and IIIA where TRP-213 residue is housed. The Protein-surfactant interactions include electrostatic, van der Waals and hydrophobic interactions. In the bound state, hydrogen-bonding interaction with the BSA existed for SDS, HPS and OG, while the CTAB head-group atoms were shielded from intermolecular hydrogen bonding. Hydrogen bonds formed by SDS and HPS with the BSA were found weaker than those of OG. However, the decomposed energy from MD simulation showed higher coulomb interaction for the ionic surfactants when compared with the non-ionic OG, and this was attributed to higher concentration of atomic partial charges in the ionic surfactants head-groups as calculated by DFT. On the other hand, the van der Waals energy was found to scale directly with the size of hydrophobic alky tail and showed no trend with the amount of partial charge on the surfactants head- or tail-groups. Interestingly, DFT calculations revealed that the surfactants tail-groups are not completely uncharged. The existence of some amount of partial charge on the free and bound surfactants tail-groups observed in this study provides an important means of characterization of surfactant aggregation and micelle core polarity in bulk solutions, which are the most difficult to model experimentally.

The surfactants binding free energies which were calculated by LIE approach agreed well with experimental values. The calculated binding free energies were dominated by differences between the van der Waals energies in ligand-bound and free states. These findings present a way of optimizing protein-surfactant binding interactions which is advantageous for studies on surfactant-induced protein stability or denaturation.

## Supplementary information

Supporting information
