# Author Correction: In Silico Characterization of the Binding Modes of Surfactants with Bovine Serum Albumin

**DOI:** 10.1038/s41598-020-63857-0

**Published:** 2020-04-17

**Authors:** Osita Sunday Nnyigide, Sun-Gu Lee, Kyu Hyun

**Affiliations:** School of Chemical and Biomolecular Engineering, Pusan National University, Busan, 46241 Korea

Correction to: *Scientific Reports* 10.1038/s41598-019-47135-2, published online 23 July 2019

This Article contains errors in the Methods section under subheading ‘Final molecular docking procedure’.

“AutoDock Vina generates different ligands conformers using a Lamarkian genetic algorithm (LGA). The GA is implemented with an adaptive local method search”

should read:

“AutoDock Vina generates different ligands conformers using a Broyden-Fletcher-Goldfarb-Shanno (BFGS) algorithm. The BFGS is implemented with an iterated local method search”

