# Supplementary material for: In Silico Characterization of the Binding Modes of Surfactants with Bovine Serum Albumin
Source: Sci Rep. 2019 Jul 23;9:10643. doi: 10.1038/s41598-019-47135-2 (PMC6650617; doi:10.1038/s41598-019-47135-2)
Supplement: Supplementary file 1 — Supporting information [file 41598_2019_47135_MOESM1_ESM.docx]

**In Silico Characterization of the Binding Modes of Surfactants with Bovine Serum Albumin**

Osita Sunday Nnyigide, Sun-Gu Lee* and Kyu Hyun*

School of Chemical and Biomolecular Engineering, Pusan National University, Busan 46241, Korea

**Supporting Information**

*Correspondence to sungulee@pusan.ac.kr or kyuhyun@pusan.ac.kr

Table **S1**. Training set for the estimation of β and α parameters using Experimental and LIE binding free energies (kJ/mol) of various BSA-surfactant complexes^a^.

| Ligand | $\boldsymbol{\Delta G}_{\boldsymbol{bind, exp}}$ [kJ/mol] | $\boldsymbol{\Delta G}_{\boldsymbol{bind, lie}}$ [kJ/mol] |
| --- | --- | --- |
| Dodecyl glucoside (DG) | -19.46±0.46^1^ | -19.41±3.50 |
| Sodium tetradecyl sulfate (STDS) | -35.23^2^ | -31.20±4.40 |
| Sodium dodecyl benzylsulfonate (SDBS) | -34.1±2^3^ | -31.10±3.20 |
| Penta (ethylene glycol) monododecyl ether (C12E5) | -24.8±5^3^ | -22.30±3.10 |
| Tetradecyltrimethylammonium chloride (TTAC) | -25.90^4^ | -24.30±3.40 |

^a^STDS was determined at pH of 5.6, however values did not change for pH 4.8-6.8 according to the authors^2^, while TTAC was averaged over values determined at pH 5.8 (23.82 kJ/mol) and pH 8.1 (27.97 kJ/mol)^4^.


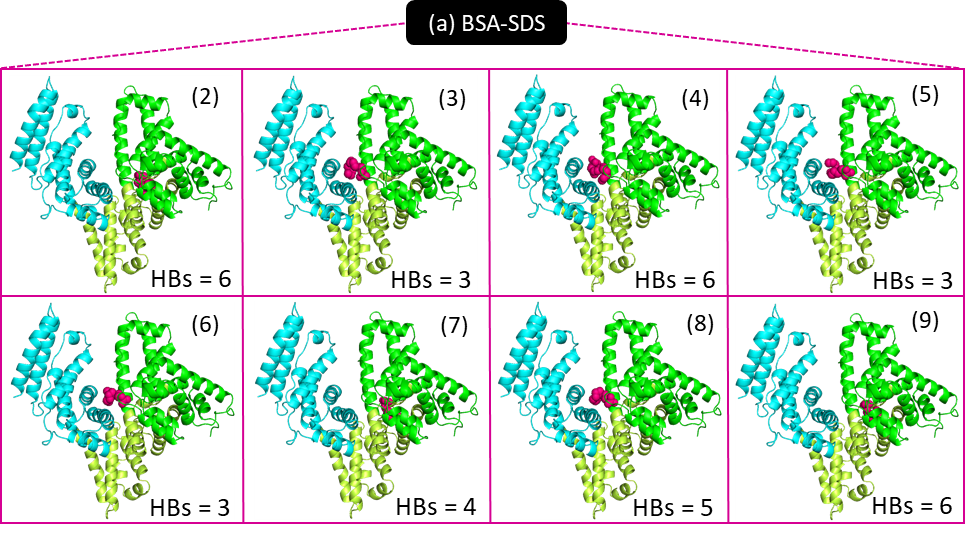


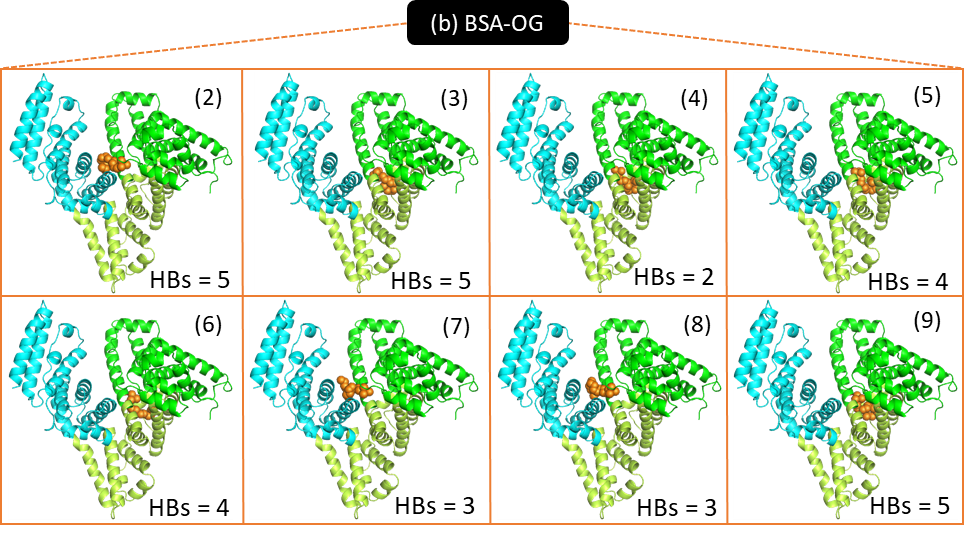


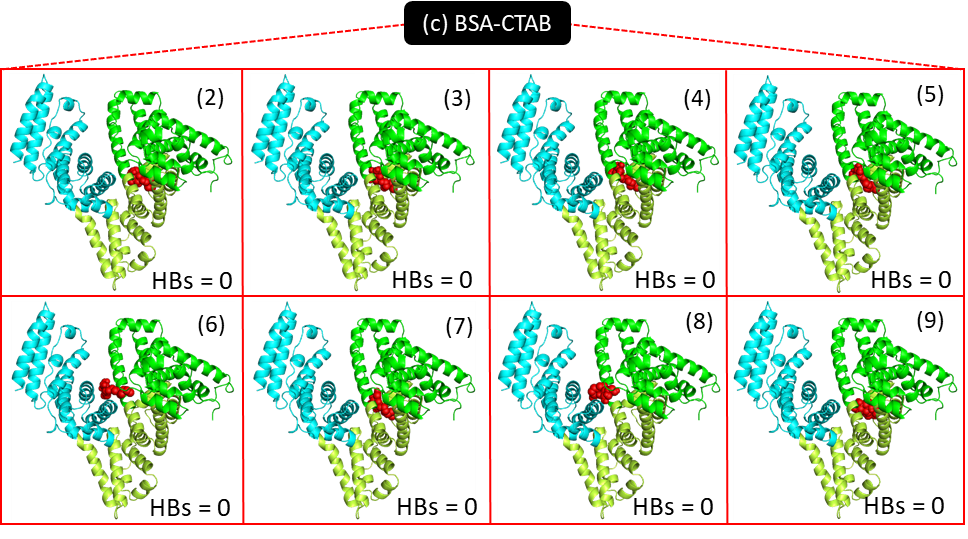


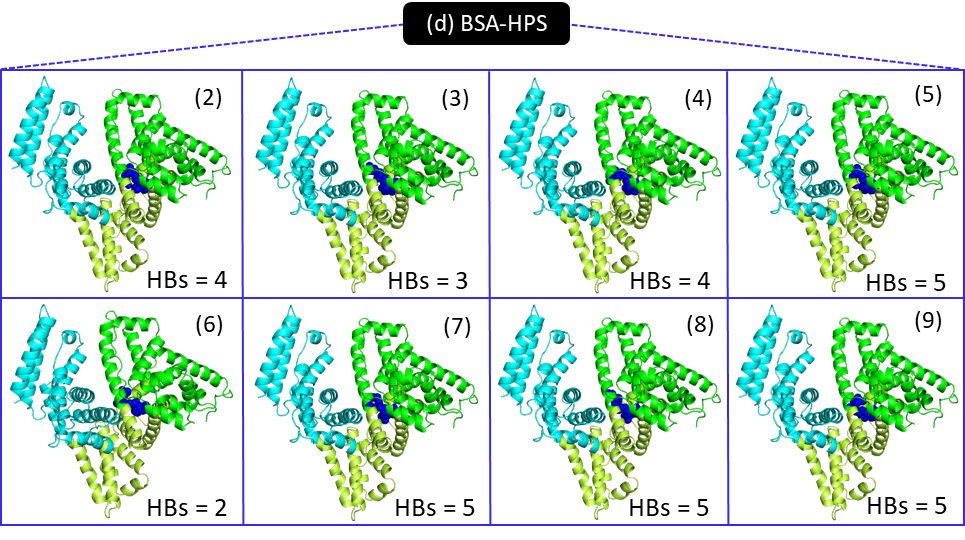


Figure **S1**. Binding conformations showing docking rank from 2 to 9 (a) BSA-SDS (b) BSA-OG (c) BSA-CTAB and (d) BSA-HPS. BSA is shown in cartoon and surfactant in sphere representations.

Table S**2**. Comparison of the top two ranked docked complexes. Number of hydrogen bonds (HBs) was taken from the docked structure and the total energy (coulomb + Van der Waals) was taken from MD simulation.

| BSA-surfactant complex | Docking rank | Total no. of HBs (docking) | Total potential energy  (kJ/mol) |
| --- | --- | --- | --- |
| BSA-SDS | 1 | 7 | -790.45 |
|  | 2 | 6 | -688.12 |
| BSA-OG | 1 | 5 | -372.77 |
|  | 2 | 5 | -324.93 |
| BSA-CTAB | 1 | 0 | -450.61 |
|  | 2 | 0 | -441.92 |
| BSA-HPS | 1 | 5 | -718.02 |
|  | 2 | 4 | -508.10 |

Table **S3**. Estimated $\left\langle U_{l-s}^{el} \right\rangle_{f}$ and $\left\langle U_{l-s}^{vdw} \right\rangle_{f}$ according to Eq. 1

| Free ligand in water | $\left\langle\boldsymbol{U}_{\boldsymbol{l-s}}^{\boldsymbol{vdw}} \right\rangle_{\boldsymbol{f}}$ | $\left\langle\boldsymbol{U}_{\boldsymbol{l-s}}^{\boldsymbol{el}} \right\rangle_{\boldsymbol{f}}$ |
| --- | --- | --- |
| CTAB | -147.75±2.60 | -236.20±2.40 |
| SDS | -87.25±1.30 | -676.65±3.60 |
| HPS | -150.07±1.00 | -585.11±5.80 |
| OG | -75.07±1.40 | -321.30±4.70 |

**REFRENCES**

1. Wasylewski, Z. & Kozik, A. Interaction of Bovine Serum Albumin with Alkyl Glucosides Studied by Equilibrium Dialysis and Infrared Spectroscopy Eur. J. Biochem. **95**, 121 – 126 (1979).
2. Reynolds, J.A., Gallagher, J.P. & Steinhardt, J. Effect of pH on the binding of N-alkyl sulfates to bovine serum albumin. Biochemistry, **9**, 1232-1238 (1970).
3. Nielsen, A.D., Borch, K. & Westh, P. Thermochemistry of the specific binding of C_12_ surfactants to bovine serum albumin. Biochimica et Biophysica Acta. **1479**, 321-331 (2000).
4. Nozaki, Y., Reynolds, J. & Tanford, C. The interaction of a cationic detergent with bovine serum albumin and other proteins. J. Biol. Chem. **249**, 4452-4459 (1974).
